# Seed-Assisted Synthesis of Graphene Films on Insulating Substrate

**DOI:** 10.3390/ma12091376

**Published:** 2019-04-28

**Authors:** Qiqi Zhuo, Yipeng Mao, Suwei Lu, Bolu Cui, Li Yu, Jijun Tang, Jun Sun, Chao Yan

**Affiliations:** 1 College of Material Science & Engineering, Jiangsu University of Science and Technology, 2 Meng-Xi Road, Zhenjiang 212003, Jiangsu, China; zqq88263268@just.edu.cn (Q.Z.); maoyipeng@stu.just.edu.cn (Y.M.); 152210603127@stu.just.edu.cn (S.L.); 152210603101@stu.just.edu.cn (B.C.); 152210603106@stu.just.edu.cn (L.Y.); 200600002479@just.edu.cn (J.T.); 2 College of Chemistry, Chemical Engineering and Material Science, Soochow University, 199 Ren-Ai Road, Suzhou 215123, Jiangsu, China; sunjun@suda.edu.cn

**Keywords:** graphene, insulating substrate, graphene oxide, seeds

## Abstract

Synthesizing graphene at a large-scale and of high quality on insulating substrate is a prerequisite for graphene applications in electronic devices. Typically, graphene is synthesized and then transferred to the proper substrate for subsequent device preparation. However, the complicated and skilled transfer process involves some issues such as wrinkles, residual contamination and breakage of graphene films, which will greatly degrade its performance. Direct synthesis of graphene on insulating substrates without a transfer process is highly desirable for device preparation. Here, we report a simple, transfer-free method to synthesize graphene directly on insulating substrates (SiO_2_/Si, quartz) by using a Cu layer, graphene oxide and Poly (vinyl alcohol) as the catalyst, seeds and carbon sources, respectively. Atomic force microscope (AFM), scanning electronic microscope (SEM) and Raman spectroscopy are used to characterize the interface of insulating substrate and graphene. The graphene films directly grown on quartz glass can attain a high transmittance of 92.8% and a low sheet resistance of 620 Ω/square. The growth mechanism is also revealed. This approach provides a highly efficient method for the direct production of graphene on insulating substrates.

## 1. Introduction

Synthesizing large-scale and high quality graphene is a prerequisite for graphene applications in electronics [1,2,3,4,5]. Since the first mechanical exfoliation from highly oriented pyrolytic graphite, various methods to synthesize graphene have been developed, including the chemical reduction of graphene oxides [6,7,8,9,10,11], chemical vapor deposition (CVD) [12,13,14,15,16,17], and epitaxial growth on SiC substrate [18,19]. Among those methods, graphene synthesized by CVD on metal substrates, such as Cu [20,21], Ni [22,23], and Pt [24,25] has unique advantages for the formation of graphene over a large area and of high quality. However, CVD-grown graphene film is necessary to transfer to insulating substrates such as polyethylene terephthalate (PET) and SiO_2_, for optoelectronic and electronic applications. This prominent drawback is also inevitable for the metal-CVD method, which limits its further development and reduces its superiority. The complicated transfer process of chemical etching or electrochemical bubbling involves some issues such as surface wrinkles, residual contamination and the breakage of graphene films, which will greatly degrade its performance [26,27]. Hence, great efforts have been focused on developing alternative preparation methods that can avoid the transfer process. For instance, Tang et al. [28] directly synthesized a single-crystalline graphene grain of 20 µm in size on hexagonal boron nitride (h-BN). Both the carrier mobility and the grain size were among the best for CVD-grown graphene. Weber et al. [29] synthesized graphene films on various insulating substrates such as SiO_2_/Si, Al_2_O_3_ and quartz glass by low pressure chemical vapor deposition (LP-CVD) using CO_2_ as oxidant and CH_4_ as the carbon source. The obtained graphene films show a good electrical transport property with a charge carrier mobility of 720 cm^2^ V^−1^ S^−1^. In addition, the direct growth of graphene on insulating substrates such as SiO_2_ [30,31], Al_2_O_3_ [32,33], quartz [34,35] and BN [36,37] by CVD have also been demonstrated. This method requires a long thermal annealing time or a high temperature. Moreover, it is difficult to obtain graphene films of large size and high quality. A method for the rapid growth of only a few layers of graphene on insulating substrates remains necessary for optical and electronic applications.

According to the carbon solubility in the metal substrates, the mechanisms of graphene growth on metals can be divided into two categories [38,39,40,41,42,43]. For graphene grown on metals that have large carbon solubility such as Fe, Ni, Co and Ru, a dissolution-segregation-precipitation mechanism is recognized, which is usually used to synthesize multilayered graphene. And for metals with a relatively lower carbon solubility such as Cu, a surface growth mechanism is recognized. This method is usually used to synthesize single or a few layers of graphene. During the growth process, carbon atom nucleation is considered to be a critical factor for graphene growth. In order to accelerate or control the orientation of graphene growth, several works have used pre-seed as the nucleation to growth graphene. For example, Wei and Wee et al. [44] used peel-off graphene and the graphene nanocluster as the seeds for graphene growth. Graphene prepared by this method proves to be as high quality as mechanically exfoliated graphene. Except for peel-off graphene, GO [45,46], PMMA [47,48] and coronene [49] and so forth, also can be used as nucleation seeds for graphene synthesis.

Here, we report a facile method to synthesize uniform and large-area graphene directly on insulating substrates (SiO_2_/Si, quartz) using Cu, GO (graphene oxide), and PVA (polyvinyl alcohol) as catalyst, seeds, and carbon sources, respectively. Atomic force microscope (AFM), scanning electron microscope (SEM) and Raman spectroscopy are used to characterize the formation process of graphene, also a tentative mechanism is presented to describe the growth processes for graphene films.

## 2. Materials and Methods 

### 2.1. Materials

PVA, H_2_SO_4_, H_2_O_2_, HCl and CuSO_4_ were purchased from Sinopharm Chemical Reagent, China. GO was prepared from natural graphite powder using the modified Hummers method. Deionized water with a resistivity of 18.1 MΩ cm was used for all experiments.

### 2.2. Preparation of GO Seeds

As-prepared GO was dispersed into de-ionized water under ultrasonic treatment to obtain a uniform GO aqueous solution (0.1 mg/mL). Afterwards, the GO aqueous solution was processed by centrifuging and the supernatant were collected and used as seeds for graphene synthesis. Different sizes of GO seeds can be collected at different centrifuging speeds. GO with the size of ~260 nm is adopted in this research.

### 2.3. Preparation of PVA Aqueous Solution

0.1 g PVA powder was first added to 10 mL de-ionized water with magnetic stirring at room temperature for 2 h and was then dissolved at 90 °C for 4 h to form 1 wt % PVA solutions.

### 2.4. Preparation of PVA Aqueous Solution

First, SiO_2_/Si wafer with 300 nm SiO_2_ in thickness was cleaned by ethanol, acetone, and de-ionized water respectively, then treated with Piranha solution (H_2_SO_4_:H_2_O_2_ = 3:1) at 90 °C for 30 min. Afterwards, GO seeds and PVA aqueous solution were spin-coated on the SiO_2_/Si wafer sequentially. Then 200 nm Cu and 100 nm CoO layers were deposited sequentially by magnetron sputter (Kurt J. Lesker, PVD75). After annealing in CVD furnace, graphene was synthesized between the Cu layer and the insulating substrate. At last, Cu and CoO layer were etched away by Marble’s reagent (CuSO_4_:HCl:H_2_O = 10 g:50 mL:50 mL), then graphene was obtained directly on SiO_2_ substrate without any transfer process. The process can be duplicated on other insulating substrates such as quartz glass to directly grow graphene.

### 2.5. Characterizations

The size of GO was measured by Zeta-Size (ZEN3690, Malvern, Malvern, UK). GO aqueous solution was placed in disposable cuvettes and the dynamic scatter intensity was recorded at 25 °C. Raman spectra images of graphene films were obtained by a Jobin-Yvon HR800 Raman spectrometer with 633 nm wavelength incident laser at room temperature, spot size was 1 μm. The morphology of the products was observed by SEM (FEI Quanta-200) and an optical microscope (Nikon LV100D, Nikon, Tokyo, Japan). The thickness of the graphene was measured by atomic force microscopy (AFM) (Veeco MultiMode V, Billerica, MA, USA) under tapping mode with a resonance vibration frequency of 350 kHz and a scanning rate of 0.998 Hz. The electrical conductivity of graphene films was measured using a digital four-point probe system (SZ-2258, Jingge Electronic, Suzhou, China). The final result was the average of data from five devices.

## 3. Results and Discussion

### 3.1. Synthetic Process of Graphene

**Figure 1:**
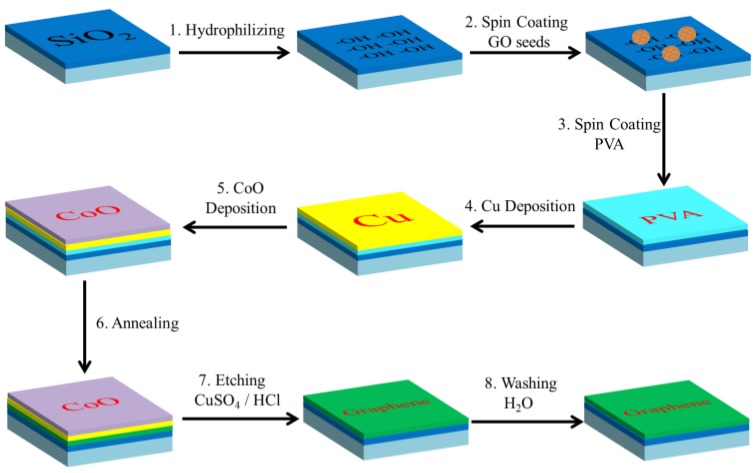
Synthesizing graphene directly on SiO_2_/Si without transfer.

**Figure 2:**
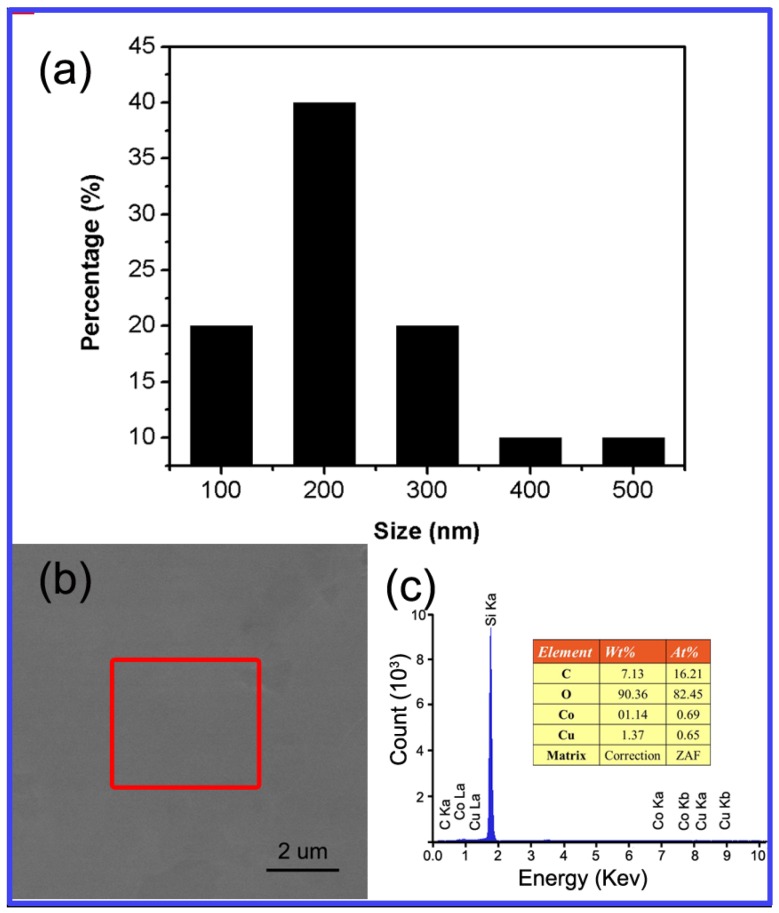
(**a**) Size distribution of GO seeds by centrifuging speedo 20000 rpm. (**b**) The SEM image of sample surface after Cu and CoO layers are etched. (**c**) The corresponding EDX data of the remarked area in (**b**).

Figure 1 illustrates the schematic of the direct growing process of graphene on SiO_2_/Si substrate. After treating with H_2_O_2_/H_2_SO_4_ (1/3) solution, hydroxyl groups were formed on the surface of the SiO_2_/Si substrate, which makes them hydrophilic. Then, GO seeds and PVA aqueous solution are spin-coated to it sequentially. Figure 2a shows the size distribution of GO seeds collected at centrifuging speed centrifuging speedo 20000 rpm. Due to hydrogen bond and Van der Waals force between GO, PVA and hydroxylation SiO_2_ substrate, GO sheets and PVA are tightly absorbed to the surface of SiO_2_ substrate. After that, Cu and CoO layers are sequentially deposited on it by magnetron sputter. Cu layer can catalyze PVA to decompose to supply the carbon source for graphene growth and CoO layers can efficiently prevent Cu from vaporizing at high temperatures [50]. Then graphene is synthesized between the Cu layer and the insulating substrate by annealing in the CVD furnace. After Cu and CoO layer are etched away, the graphene film is finally obtained directly on the SiO_2_ substrate without any transfer process. The Energy Dispersive X-ray (EDX) test (Figure 2b,c) shows there is little Cu (0.69 at%) or Co (0.65 at%) residuals left on graphene films. 

### 3.2. Structural and Morphological Investigations

**Figure 3:**
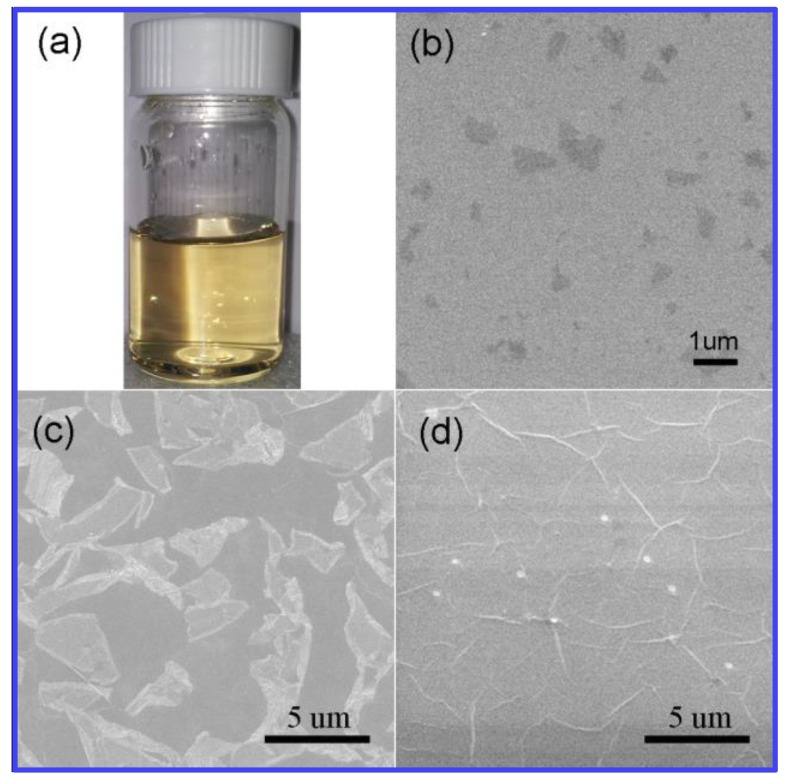
Graphene grown by GO and PVA. (**a**) GO seed aqueous solution. (**b**–**d**) SEM images of graphene grown by GO and PVA at 1000 °C for 0, 5, 15 min, respectively.

**Figure 4:**
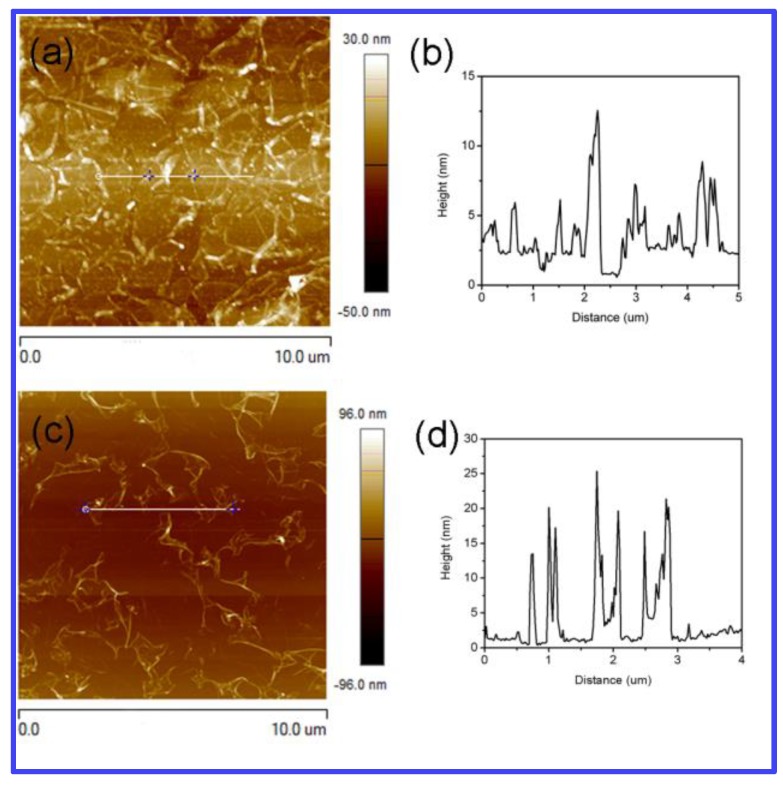
(**a**) AFM image of graphene by PVA with GO seeds. (**b**) The corresponding height curve; (**c**) AFM image of graphene grown by PVA without GO seeds after 15 min at 1000 °C and the corresponding height curve (**d**).

GO sheets with an average size of ~260 nm are adopted in this research (Figure 3a). Figure 3b shows the SEM image of GO sheets by spin-coating on a SiO_2_ substrate. The dangling bonds on the edge of the GO sheets give the marginal area high activity, so GO as seed can easily induce the deposition of carbon atoms and the growth of graphene. During the process, PVA used as a carbon source is decomposed to help the graphene grains grow up. Growth time is found to play a critical role in the morphology of graphene films grown by the present method. As shown in Figure 3c, the size of graphene grains are 3–10 μm for 5 min at 1000 °C. As time goes on, the grains can finally form a continuous graphene film at 15 min (Figure 3d), in which the grain boundary is clear to see. Figure 4a,b shows the typical AFM height images of graphene synthesized by GO and PVA. It is obvious to see the thickness increase at the edge of the grain boundary. In order to study the role of GO seeds in the growing process, graphene is also synthesized by PVA without GO as seeds in the same annealing conditions. The AFM image in Figure 4c showed that no continuous graphene film can be formed by PVA without GO seeds. The corresponding height curve in Figure 4d demonstrated there are many wrinkles on the surface of synthesized film.

**Figure 5:**
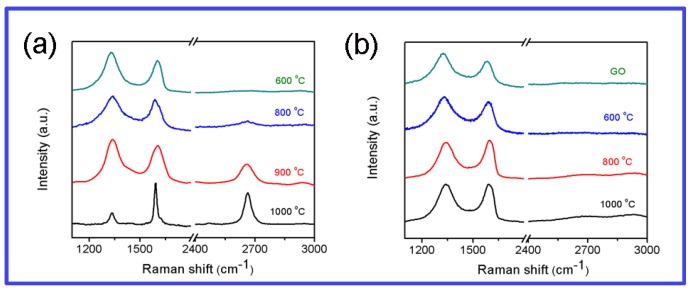
(**a**,**b**) Raman spectra of products synthesized by PVA with GO and without GO seeds at different temperature.

The quality of products on insulating substrates was evaluated by using Raman spectroscopy with laser excitation at 633 nm. The G band at 1570–1600 cm^−1^ is attributed to the first-order scattering of E_2g_ mode, and the D band at about 1330 cm^−1^ arises from a breathing mode of κ-point phonons of A_1g_ symmetry. The D band is related to the amount of disorder and its intensity reflects the degree of defects. The 2D band originates from a two phonon double resonance Raman process. Thus, the intensity ratio of D and G bands(I_D_/I_G_) of carbon material indicates the degree of the disorder such as defects, edges and ripples [6]. Figure 5a shows Raman spectra of products synthesized by PVA with GO seeds at different temperatures. With the growth temperature increasing from 600 °C to 1000 °C, the ratio of I_D_/I_G_ decreased gradually from 1.23 to 0.31. Meanwhile the 2D (2662 cm^−1^) band appears, indicating that the graphene is of high quality. This may be attributed to the formation of conjugated carbon atoms and the removal of the oxygenous groups from GO. The intensity ratio of G and 2D bands (I_G_/I_2D_) as well as the full width at half maximum (FWHM) of the 2D peak were usually used to indicate the number of graphene layers. The I_G_/I_2D_ value of 0.78 and 2D peak at 45 cm^−1^ from FWHM demonstrate the prepared graphene film was of bilayer [51,52]. Figure 5b shows Raman spectra of graphene synthesized by PVA without GO seeds at different annealing temperature. Broad peaks occurred around 1330 (D band) and 1590 cm^−1^ (G band) but no peaks around 2660 cm^−1^ (2D bands). These results indicate that without GO seeds, PVA is decomposed and only converted to amorphous carbon. 

**Figure 6:**
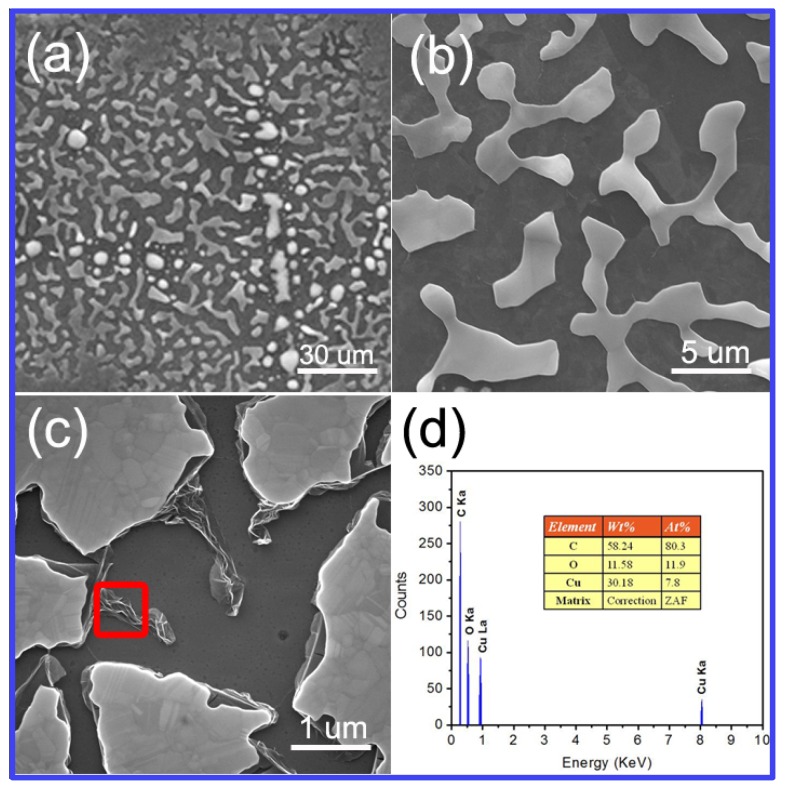
(**a**–**c**) Different magnified SEM images of the Cu film without CoO layer after annealing. (**d**) EDX of remark area in **c**.

During the annealing process, CoO layers can efficiently prevent Cu from vaporizing at a high temperature and promote the growth of a continuous graphene film. Figure 6a–c shows different magnified SEM images of Cu film without CoO layers after annealing. The Cu layer is evaporated partly to form an isolated island structure and the wrinkled graphene film can be observed at the edge of the Cu film. The energy dispersive X-ray (EDX) spectroscopy demonstrates that the main ingredient of the films is carbon (Figure 6d). The wrinkled graphene may be formed due to the stress-induced graphene sheet rupture and new graphene nucleate subsequently at the ruptured sites during the Cu reducing process [53]. On the contrary, the surface of graphene with CoO layers is uniform and continuous after annealing.

**Figure 7:**
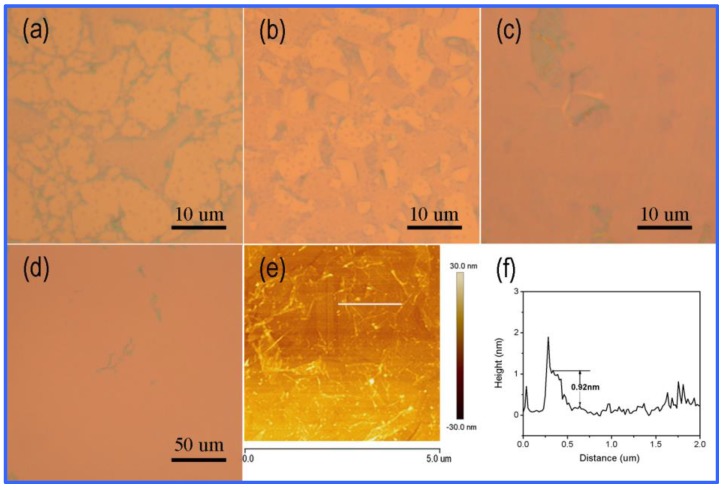
(**a**–**d**) Optical images of graphene grown by GO and PVA at 1000 °C for 2 min (**a**), 5 min (**b**), 15 min (**c**,**d**), respectively. (**e**) AFM image of graphene by PVA with GO seeds after 15 min. (**f**) the corresponding height curve.

Based on the different refractive index between graphene and SiO_2_ [54,55], an optical microscope is also used to investigate the growth process of graphene films. Figure 7a–d show the optical images of graphene grown by GO and PVA at 1000 °C for different times. Figure 7e shows the typical AFM height images of graphene synthesized by GO and PVA. It’s obvious to see the thickness increase at the edge of grain boundary. The thickness of the graphene film (Figure 7f) is about 0.92 nm, suggesting that the graphene is bilayer, which is in accordance with the Raman spectra results. Compared to CVD transferred graphene, graphene directly grown on insulating substrate shows less wrinkles, contamination and breakage.

### 3.3. Mechanism of Graphene Growth and Ultraviolet-Visible Spectra

**Figure 8:**
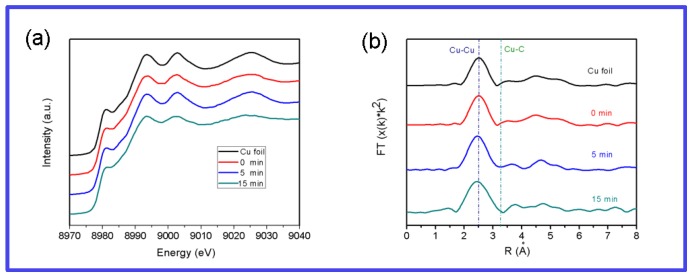
In situ XAFS of Cu foil, Cu/GO/PVA (**a**) and its corresponding Fourier transform magnitudes (**b**) at different time, respectively.

To clarify the mechanism of graphene growth with GO/PVA, in-situ X-ray absorption fine structure spectroscopy (XAFS) was used to detect the chemical state and the local structure of the copper layer during the growth process. In Figure 8, the XAFS spectra of the initial Cu, Cu/GO/PVA are shown. The chemical valence state of Cu for Cu/GO/PVA is similar to that of Cu foil and no obvious change can be found at a different time. Also, no peak arises at 3.2 Å indicating no Cu-C alloy is formed during the process. The result directly demonstrates that the growth of graphene only occurs on the Cu surface. 

**Figure 9:**
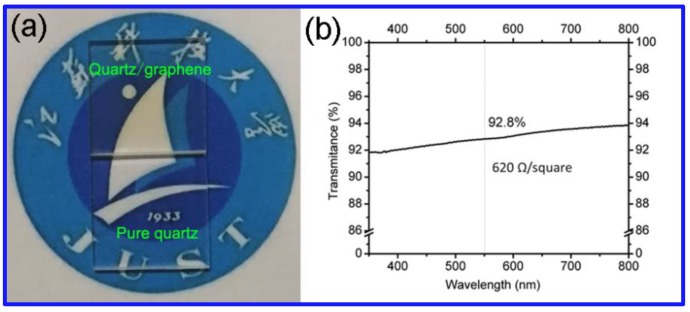
(**a**) Optical images of graphene grown on quartz glass and pure quartz glass. (**b**) Ultraviolet-visible spectra of the CVD graphene films on quartz glass.

Due to its excellent transmittance and electrical conductivity, graphene is considered to be an ideal material to replace indium tin oxide (ITO) as transparent electrodes. However, the complex transfer process of graphene to transparent substrate usually introduces a variety of defects into the graphene sheet. The present method should provide a possible route to directly grow graphene on transparent substrates without the need for any transfer process. In this research, graphene is also directly fabricated on quartz glass. Figure 9 shows the optical images and UV spectra of graphene grown on quartz glass. The thin graphene films are macroscopically smooth and uniform. Compared with other methods, graphene on the quartz glass substrate can exhibit a 92.8% optical transmittance and the lowest square resistance (620 Ω/square) by our method. 

**Figure 10:**
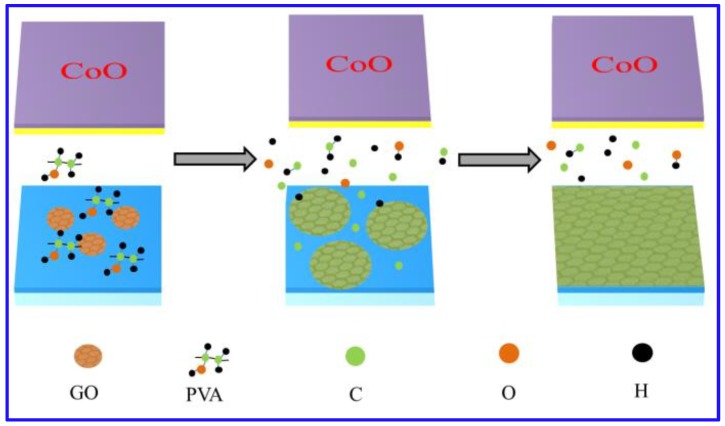
The mechanism of synthesizing graphene directly on insulating substrates without transfer.

On the basis of those above-mentioned observations, a tentative mechanism is presented to describe the growth processes for graphene films, as shown in Figure 10: (1) GO seeds and PVA are deposited on the insulating substrate sequentially. GO resembles the basic structure of graphene due to containing cyclobenzenes and it has high activity with dangling bonds on the edge of GO sheets, it also offers a reasonable nucleus size for graphene growth. PVA with lower bond dissociation energy of C-C single bonds can be used as a carbon source for graphene growth. Also, both GO and PVA can be dispersed homogeneously on the hydrophilizated substrate due to containing function groups such as hydroxyl, carbonyl and carboxy; (2) The annealing process mainly includes following steps: (i) PVA decompose to generate carbon atom under the catalysis of Cu in hydrogen atmosphere; (ii) GO is reduced to form r-GO; and (iii) Carbon atoms are deposited around r-GO and as seeds for graphene grains growth; (3) Grains grow up and coalesce to from continuous graphene films. Below are the chemical equations:

(1) $${\left[C_{2}H_{4}O\right]}_{n}\overset{\mathrm{Cu}/H_{2}}{\underset{\Delta }{\to }}C\cdot +H\cdot +O$$

(2) $$\mathrm{GO}\overset{\mathrm{Cu}/H_{2}}{\underset{\Delta }{\to }}\mathrm{r-GO}+O$$

(3) $$\mathrm{r-GO}+C\cdot \overset{\mathrm{Cu}/H_{2}}{\underset{\Delta }{\to }}\mathrm{Graphene}$$

Table 1 compares the present method quantitatively with other techniques that have been reported for growing graphene directly onto a substrate. Our method appears to be one of the most attractive routes towards a controllable transfer-free route for graphene growth.

## 4. Conclusions

In summary, we have developed a transfer-free method for the large-scale synthesis of graphene on insulating substrate using a Cu layer, GO and PVA as the catalyst, seeds and carbon sources, respectively. During the growth process, GO seeds can act as the nucleus to efficiently help the graphene films to form. Raman spectra shows a ratio of I_D_/I_G_ was 0.31 at 1000 °C, meanwhile the 2D band appears, indicating that the graphene is of relatively high quality. By this method, continuous graphene films can be prepared on quartz glass and used as transparent electrodes, which showed optical transmittance of 92.8% and resistances of 620 Ω/square. XAFS study shows that the growth of graphene only occurs on the Cu surface following the surface-mediated nucleation mechanism. Further work must be performed in order to evaluate the electronic transport performance of this material and therefore validate its use in practical applications like solar cells or organic light-emitting diodes.

## Figures and Tables

**Table 1:** Comparison of measured parameters of graphene prepared through different methods.

Substrate	Size(mm)	Raman (ID/IG)	Transmittance (%)	Square Resistance (KΩ/sq)	Ref.
Quartz	Limit by substrate	~1	92.0	0.9	[56]
Quartz	~200	~0.2	82.0	0.27	[57]
SiO_2_	Limit by substrate	<0.2	N/A	N/A	[58]
Quartz	10	~1	84.0	30	[59]
Quartz	N/A	~2	~70	0.6	[60]
SrTiO_3_	Limit by substrate	~1	96.4	0.95	[61]
Quartz	Limit by substrate	0.31	92.6	0.62	this paper
